# The Gut Microbiome and Diet Interact to Dictate Personalized Response to Broccoli Sprout Consumption

**DOI:** 10.1002/fsn3.72211

**Published:** 2026-08-05

**Authors:** Alexandra Alexiev, Laura M. Beaver, John A. Bouranis, Carmen P. Wong, Jan F. Stevens, Thomas J. Sharpton, Emily Ho

**Affiliations:** ^1^ Department of Microbiology Oregon State University Corvallis Oregon USA; ^2^ Linus Pauling Institute, Oregon State University Corvallis Oregon USA; ^3^ School of Nutrition and Public Health Oregon State University Corvallis Oregon USA; ^4^ Department of Pharmaceutical Sciences Oregon State University Corvallis Oregon USA; ^5^ Department of Statistics Oregon State University Corvallis Oregon USA

**Keywords:** broccoli sprouts, diet, gut microbiome, personalized nutrition

## Abstract

Broccoli sprouts produce sulforaphane (SFN), a dietary isothiocyanate with anti‐cancer and anti‐inflammatory properties. Human intervention trials observe large variation in metabolite generation and bioactivity with broccoli sprout consumption across individuals. We hypothesize that interactions between pre‐intervention diet and personalized gut microbiome composition contribute to this variation. This exploratory analysis leverages a small existing dataset to test novel mediation pathways, with the goal of identifying associations for future validation in larger, prospectively designed studies. In a trial of 38 healthy adults, we analyzed participant‐recorded 7‐day food diaries, profiled baseline gut microbiome compositions, and quantified urinary SFN metabolites over 72 h after broccoli sprout consumption. Using regression‐based mediation analysis, we modeled two complementary predictions: (1) that specific gut bacterial genera mediate how pre‐intervention diet composition influences SFN metabolism, and (2) that particular dietary components mediate how gut microbiome composition influences SFN metabolism. These analyses found that seven genera, including *Collinsella, Ruminococcus*, and *Bifidobacterium*, mediated relationships between pre‐intervention diet and SFN metabolites, particularly the bioactive forms. Unsupervised clustering further revealed three distinct baseline gut microbiome community “types,” each exhibiting differential production of SFN‐nitrile, a biologically inert metabolite, with effects mediated by the consumption of specific carbohydrate classes. Together, these findings indicate that microbiome structure, in concert with diet, shapes individual SFN metabolic outcomes. Considering pre‐intervention diet and gut microbiome may therefore enhance the design and personalization of cruciferous vegetable interventions.

## Graphical abstract

The study investigated how diet, the gut microbiome, and their interactions influence the production of different types of sulforaphane metabolites, specifically bioactive (have health benefits) and inert (do not have health benefits). Mediation analysis explored these tripartite relationships.

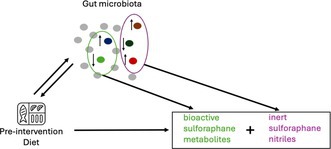

**Abbreviations**
ACME: average causal mediation effect
dbRDA: distance‐based redundancy analysis
IBD: inflammatory bowel disease
MSE: mean squared error
PERMANOVA: permutational multivariate analysis of variance
SFN: sulforaphane
SFN‐CG: sulforaphane cysteine‐glycine
SFN‐Cys: sulforaphane‐cysteine
SFN‐GSH: sulforaphane‐glutathione
SFN‐NAC: sulforaphane‐N‐acetyl‐cysteine
SFN‐NIT: sulforaphane‐nitrile

## 1 Introduction

Dietary intake and habits strongly influence health outcomes and disease risk, particularly for prevalent chronic conditions such as diabetes, cardiovascular disease, and cancer (Beaver et al. 2018; Bonnesen et al. 2001; Curioni et al. 2022; Neuhouser 2019). One mechanism by which diet affects health is by shaping the microbiome, particularly through dietary compounds such as pre‐biotic food components like fiber (Carmody et al. 2024; David et al. 2014; Ley et al. 2008; Li et al. 2009; Muegge et al. 2011). In turn, the microbiome participates in the metabolism of food‐derived compounds (Bouranis et al. 2024; Rowland et al. 2018; Vernocchi et al. 2020), and the specific taxa present can determine which bioactive molecules are ultimately produced (Bouranis et al. 2024, 2023, 2022; Bouranis, Beaver, and Ho 2021; Rowland et al. 2018; Vernocchi et al. 2020; Clarke et al. 2011; Liu et al. 2017). In particular, intake of specific foods may modify sulforaphane (SFN) production after consumption of broccoli sprouts by altering gut microbiome composition (Li et al. 2009, 2011; Liu et al. 2017; Bouranis, Choi, et al. 2021). Fiber, in particular, has been associated with both microbiome restructuring and SFN metabolism (Li et al. 2009).

SFN is a bioactive phytochemical derived from cruciferous vegetables such as broccoli, Brussels sprouts, kale, mustard greens, and cabbage. SFN and its metabolites have been linked to reduced risk and improved outcomes in cancer, diabetes, cardiovascular disease, and neurodegenerative diseases (Beaver et al. 2018; Bonnesen et al. 2001; Atwell et al. 2015; Azarenko et al. 2014; Basten et al. 2002). Yet interindividual variability in SFN metabolism leads to a highly individualized response to broccoli sprout feeding trials—for example, Fahey et al. found that conversion of glucosinolates, such as glucoraphanin, into bioactive metabolites, ranged as much as 1.1%–40.7% in their study population (*n* = 45) (Fahey et al. 2012). Evidence from our group and others suggests that the human gut microbiome, which itself is also highly variable between individuals, influences the bioavailability and metabolism of SFN (Li et al. 2009, 2011; Bouranis et al. 2024; Bouranis, Choi, et al. 2021; Fahey et al. 2012). Although typical microbiome metrics of alpha and beta diversity do not link consistently to SFN metabolism across many of these studies, specific taxa and SFN metabolites are correlated (Bouranis et al. 2024, 2023; Li et al. 2011). Specifically, our group previously correlated whole‐community diversity measures of the gut microbiome with broccoli sprout consumption and noted inconsistent associations and marginal effect sizes (Bouranis et al. 2024, 2023). Specific taxa and SFN metabolites were correlated, however: *Ruminococcus* ASVs were negatively correlated with bioactive SFN metabolites, while others were positively correlated with these same metabolites, and many *Bifidobacterium* strains were positively correlated with SFN‐NIT production (Bouranis et al. 2024). Our group and others demonstrated that members of the gut microbiota (e.g., *Lactobacillus* and *Enterococcus*) were able to break down glucosinolates from cruciferous vegetables into different SFNs in vitro (Bouranis et al. 2023; Bouranis, Beaver, and Ho 2021; Luang‐In et al. 2016, 2014; Palop et al. 1995). The relationship between SFN metabolism and gut microbiome diversity metrics may not be consistent between studies, but the abundance of specific taxa is demonstrably linked to SFN metabolism. Interindividual variability in gut microbiome composition may therefore contribute to variability in the formation of beneficial SFN metabolites following cruciferous vegetable consumption (Li et al. 2009, 2011; Bouranis et al. 2024; Bouranis, Choi, et al. 2021; Fahey et al. 2012).

Because gut microbial abundances are shaped by host and environmental factors, chiefly diet, these interactions likely extend beyond the microbiome itself. Both overall dietary patterns (e.g., vegetarian versus omnivore) and specific dietary components such as fiber and B vitamins have been shown to influence gut microbiome composition and metabolic activity (Carmody et al. 2024; David et al. 2014; Ley et al. 2008; Muegge et al. 2011). Whole diet may influence SFN production via gut microbiome composition. Previous research found that prolonged consumption of cruciferous vegetables may “prime” the gut microbiota for broccoli sprout consumption such that more SFN metabolites are produced (Li et al. 2009, 2011; Liu et al. 2017; Bouranis, Choi, et al. 2021). Fahey, et al. found that the rate of conversion of broccoli sprout extract and the speed at which the bulk of the conversion occurred explained variation in SFN response; this was hypothesized to be related to food matrix effects on the gut microbiome (Fahey et al. 2012). Dietary fiber in particular has been associated with effects on both gut microbiome composition and SFN production (Li et al. 2009). Yet, few studies have simultaneously examined how habitual diet and the gut microbiota interact to define SFN metabolism. Here, we link all three (diet, gut microbiota, and SFN metabolism) using a regression‐based mediation framework to generate candidate causal pathways describing how pre‐intervention diet and gut microbiota may jointly shape SFN metabolic outcomes.

Our goal was to determine whether habitual diet influences the gut microbiome in ways that alter individual responses to broccoli sprout consumption, resulting in personalized SFN metabolism. To address this, we used a regression‐based mediation framework that enables exploratory causal inference while jointly modeling diet, gut microbiota, and SFN metabolites. This approach provides a follow‐up perspective to prior research that uncovered pairwise relationships between diet, microbiota, and SFN metabolism (Li et al. 2009; Bouranis et al. 2024, 2023, 2022; Bouranis, Choi, et al. 2021; Ross et al. 2024) and identifies candidate taxa for future in vitro experimentation to resolve the underlying enzymatic or metabolic mechanisms. Leveraging data from a previous human feeding trial, we implemented mediation analysis to test two predictions: (1) whether gut microbes mediate pre‐intervention diet and SFN metabolism, and (2) whether diet mediates the relationship between microbiome composition and SFN metabolism. Elucidating how diet and gut microbiota jointly shape SFN metabolism advances our understanding of interindividual variation in cruciferous‐vegetable responses and may inform strategies to optimize their health benefits.

## 2 Experimental Section

### 2.1 Study Design and Dosage Information

**FIGURE 1:**
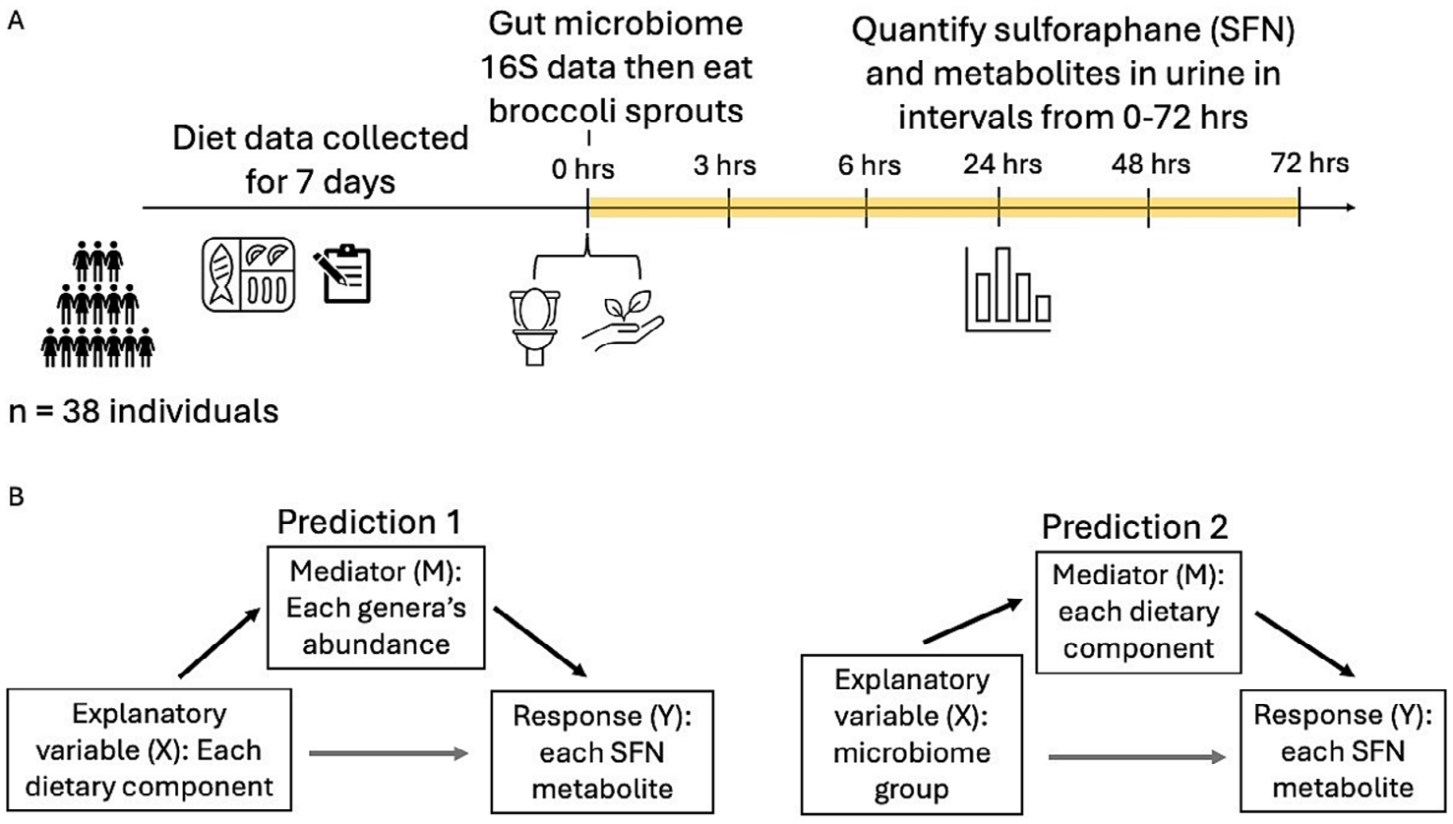
Experimental sampling design and approach to analysis. (A) Shows the sampling design and timeline. The yellow highlighted portion indicates the time intervals during which gut microbiome and SFN metabolite data were collected. (B) Is a visual representation of the regression‐based mediation models used to test predictions 1 and 2. The gray arrow from *X* to *Y* is not tested in the mediation package code in R, but is an underlying assumption, so it was tested prior to running the mediation models (Table S1; see methods for further details).

A broccoli sprout feeding trial was conducted in 38 healthy human participants recruited from Corvallis, OR, USA [ages ranging 18–51] (Figure 1A). The study took place at Oregon State University (OSU) in the Linus Pauling Institute and the Moore Family Center metabolic kitchen. The study design was approved by the OSU Institutional Review Board (IRB #8343, NCT04641026), and exclusion criteria and study activities were described in our previous publication (Bouranis et al. 2024). To summarize, for 1 week before, and throughout the three‐day study, participants were instructed to consume their normal diet with the exception of avoiding foods and beverages containing cruciferous vegetables or live/active cultures, and herbal, phytochemical, and probiotic supplements. This study design ensured that these particular dietary components did not interfere with or confound the variables of interest (SFN and microbiome), and the exclusion of some of these dietary components is unlikely to affect the consumption of other food components, like fiber or vitamin C. However, the restriction of these food components prior to the study is a caveat that future studies will need to consider to ensure our findings generalize to normal eating behaviors. They spent the first week recording all food, beverage, supplements, and medications consumed in a daily intake log, which was also used to confirm compliance with avoiding confounding food items. Dietary intake data were then analyzed using Food Processor dietary analysis software (Trustwell, Beaverton, Oregon, USA). For each nutrient/dietary component, the mean consumption over the 7 days leading up to the study was calculated. For this analysis, only dietary components that previously associated with gut microbiome metrics and taxon abundances in our prior study were analyzed, which were as follows: total insoluble fiber (g), total soluble fiber (g), total fiber (g), cholesterol (mg), saturated fat (g), alcohol (g), monosaccharides (g), disaccharides (g), beta carotene (mcg), vitamin B1 (mg), vitamin B6 (mg), folate (DFE mcg), manganese (mg), copper (mg), iron (mg), and “other” carbohydrates [total carbohydrates – (total fiber + total mono‐ and di‐saccharides)] (Bouranis et al. 2024).

Fecal material was collected by subjects into OMNIgene GUT kits (DNA Genotek, Ottawa, ON, Canada) in the evening or morning before sprouts were consumed (zero‐hour measurement). Our previous study, Bouranis et al. (2024), collected fecal material at each time interval (0, 24, 48, 72 h post‐consumption); however, we use only the zero‐hour timepoint in this analysis since it is the most relevant to the project goals (Bouranis et al. 2024). Baseline zero‐hour spot urine measurements were also collected. Participants were then fed an average of 40.5 g of 6‐day‐old fresh broccoli sprouts ( *Brassica oleracea var. italica* ), containing ~100 μmol SFN equivalents. Preparation of fecal DNA and 16S amplicon sequencing for study subjects have been previously described (Bouranis et al. 2024). Sequences are on NCBI under BioProject PRJNA953404.

Concentrations of SFN metabolites were measured in total urine collections from 0 to 72 h post broccoli sprout consumption as an indicator of the amount of SFN that was excreted in the participants (Bouranis et al. 2024). Total urine was collected at timepoint intervals of 0–3, 3–6, 6–24, 24–48, and 48–72 h post‐consumption. SFN and SFN metabolites (sulforaphane‐glutathione [SFN‐GSH], sulforaphane‐cysteine [SFN‐Cys], sulforaphane cysteine‐glycine [SFN‐CG], sulforaphane‐N‐acetyl‐cysteine [SFN‐NAC], sulforaphane‐nitrile [SFN‐NIT]) were detected using previously described methods (Bouranis, Beaver, and Ho 2021; Clarke et al. 2011). Briefly, liquid chromatography tandem mass spectrometry (LC–MS/MS) was carried out at the OSU Mass Spectrometry Center (Corvallis, OR, USA). Total SFN (Tot) was calculated by adding all SFN metabolites (i.e., SFN, SFN‐NIT, SFN‐NAC, etc.) within each timepoint. All SFN metabolite measures were summed across all timepoints to produce a single cumulative measurement for each metabolite (including total SFN).

### 2.2 Mediation Analysis

A mediation approach was used to model the relationships between the three factors, dietary components of the pre‐intervention diet (e.g., insoluble fiber, cholesterol, etc.), gut taxa, and SFN metabolites using two predictive frameworks (Figure 1B). This regression‐based approach evaluates the effect of a mediating variable (M) on the relationship between the explanatory (X) and response (Y) variables. Specifically, if the relationship of X to Y via mediator (M) is better explained than just X to Y, then M is determined to be a significant mediator, implying that M is the causal pathway through which X affects Y. The current analyses were limited to the set of dietary components and taxonomic groups found by our previous study to be significantly associated with each other, as these are of most interest in terms of future work to optimize beneficial effects of SFN (Bouranis et al. 2024). Prior to conducting mediation analysis, partial correlations between these dietary components, taxa, and SFN metabolites were performed, which verified that diet, microbiota abundances, and SFN metabolites were sufficiently related to support mediation modeling. Only combinations of dietary components and SFN metabolites were tested that were significantly associated in a linear model without the mediating variable, as this is an assumption of the mediation model that needs to be tested prior to building the full model (Table S1). AIC‐based model selection was performed across the following model types to determine the optimal model for each relationship in the mediation: (1) Gaussian, (2) Gaussian with natural‐log‐transformed taxonomic abundances and SFN metabolites, (3) Tweedie, and (4) negative binomial with a log link function. In prediction 1, we tested the relationship of each dietary component on each SFN metabolite response, mediated by each genus abundance; in prediction 2, we tested the relationship of each microbiome community cluster (see “Gut Microbiome Groups” subsection below) on each SFN metabolite response, mediated by each dietary component (Figure 1B). Models tested each combination of a dietary component, an SFN metabolite, and a genus abundances/microbiome group. Each combination of variables was looped through independently. Across both model constructs, Gaussian linear regressions using natural log transformed taxonomic abundances and SFN metabolite measures were determined to provide the best fit of the data. Average causal mediation effect (ACME) *p*‐values were subject to FDR correction using the Benjamini‐Hochberg procedure and reported in the results. To generate a tractable set of candidate associations for experimental follow‐up, we ranked the FDR‐corrected ACME *p*‐values and prioritized those falling in the lowest 5% of the distribution; the corresponding FDR‐corrected *p*‐values are reported for all prioritized models (Table S3) so that readers may apply their own thresholds, and we note where an association also meets the conventional 0.05 level. The present study resulted in slightly higher *p*‐values in mediation, likely due to being underpowered (*n* = 38) and resulting from high variation in SFN and gut microbiome sequence samples, which is not uncommon in microbiome analyses given the high interindividual variation in microbiome data (Hammer et al. 2024). This approach was chosen to balance false positives and false negatives given the small sample size and biological rationale from prior literature. These thresholds are intended to prioritize candidate associations for experimental follow‐up rather than to establish statistical significance in the classical inferential sense. Our mediation analysis approach was implemented with the package “mediation” (version 4.5.0) in R (version 4.4.0). All plots were produced with ggplot2 (version 3.5.1) with ggpubr (version 0.6.0). A record of all code used can be found on github (https://github.com/aalexiev/Broccoli_MBmediation).

### 2.3 Evaluating Myrosinase‐Like Activity in Mediating Taxa

The resulting mediating genera from prediction 1 were evaluated for the capacity to carry out myrosinase‐like activity (i.e., enzymes that convert glucosinolates in broccoli into SFN). This was done by matching the 16S sequences from significantly mediating genera to existing reference genomes in the NCBI database (RefSeq Genome Database with “Organism” set to Bacteria [taxid:2]) via nucleotide BLAST (blastn, accessed Oct. 4, 2024). The matching reference genomes with the highest percent identity, lowest *E*‐value, highest coverage, and complete or close to complete were chosen, prioritizing type strain genomes when available. Genomes were then searched for the following myrosinase‐like protein coding genes: 6‐phospho‐β‐glucosidases or β‐glycosidases (bglA, bglX, or ascB), β‐O‐glucosidases, sulfatases (bgl4 or Tp8), and epithiospecifier protein (Bouranis, Beaver, and Ho 2021; Albaser et al. 2016; Cordeiro et al. 2015; Liou et al. 2020). There is evidence that there exist unknown homologs of genes with myrosinase‐like activity in gut microbiota (Bouranis, Beaver, and Ho 2021), so mediating taxa with no known myrosinase‐like protein coding genes could still have undiscovered genes with this function.

### 2.4 Gut Microbiome Groups

Gut microbiome subtypes were identified using K‐means clustering (testing 1–5 clusters) on Euclidean distances with silhouette scores to determine the ideal number of clusters, which were found to be 3 clusters (silhouette score = 0.35; highest score; henceforth, referred to as groups A, B, and C). We subsequently used two different modeling approaches to characterize which covariates drove the diversification of these three clusters.

First, we used the ordistep function in R to apply automatic, stepwise variable selection prior to distance‐based redundancy analysis (dbRDA) visualization, with the goal of resolving the optimal set of covariates that best explains the variation in microbiome Bray–Curtis dissimilarity across samples (optimal model formula in R: Bray–Curtis dissimilarity of gut microbiome data ~ total insoluble fiber (g) + “other” carbohydrates [e.g., organic acids, pentose sugars, sugar alcohols, starches, and gums]). Permutational multivariate analysis of variance (PERMANOVA) was used to calculate significance and portion of variability explained by these variables on microbiome Bray–Curtis dissimilarity (*p*‐values FDR‐corrected; alpha = 0.05).

Second, a Gaussian linear regression model tested which participant covariates significantly (alpha = 0.05) explained microbiome subtype membership (formula in R: microbiome cluster ~ sex + age (years) + race + weight (lbs.) + self‐reported diet type (carnivore, vegetarian, etc.) + cohort + ethnicity + height (inches) + cholesterol. (mg) + saturated fat (g) + alcohol (g) + total soluble fiber (g) + monosaccharides (g) + disaccharides (g) + beta‐carotene (mcg) + vitamin B1 (mg) + vitamin B6 (mg) + folate (DFE mcg) + manganese (mg) + copper (mg) + total fiber (g) + “other” carbohydrates (g) + iron (mg)). A supplemental analysis of prediction 2 was also modeled with bacterial genera abundance as X (Table S2) but is not the main focus of the manuscript.

A Mantel test comparing Euclidean and Bray‐Curtis distance matrices confirmed significant concordance (Mantel *r* = 0.7327, *p* = 0.001), indicating that the clusters identified under Euclidean distance are consistent with community structure captured by Bray–Curtis dissimilarity.

To complement the ordination‐based characterization of microbiome groups and directly identify taxa differing in abundance between groups, we applied ANCOM (analysis of composition of microbiomes) (Mandal et al. 2015) to the two largest and most divergent groups, A and B. Group C was excluded due to its smaller sample size, which limits reliable differential abundance inference. Taxa were considered differentially abundant when detected under conservative thresholds (detection cutoff of 0.7 with a significant effect size).

### 2.5 Modeling of SFN‐NIT Associated Taxa

We were interested in determining which bacterial genera were associated with SFN‐NIT response. First, a linear regression‐based random forest algorithm was used (randomForest package [version 4.7‐1.2] in R) as a feature filtering tool to reduce the number of taxa we needed to test with a subsequent Gaussian linear regression. The random forest model was trained on a random subset of 70% of the original data, with the remaining 30%. The final model used 105 trees to produce the lowest mean squared error (MSE) across sampled trees. Taxa with the absolute value of importance (percent increase in MSE) greater than 1 were graphed. The significance of these taxa was then assessed with a Gaussian linear regression model using the *lm* function in R (using cumulative SFN‐NIT over time as the response variable and ln(taxon abundance + 1) as the predictor) to measure the association between the abundance of the top 3 taxa and SFN‐NIT.

## 3 Results

### 3.1 Prediction 1: Gut Microbiota Mediate the Relationship Between Pre‐Intervention Diet and SFN Metabolites

**FIGURE 2:**
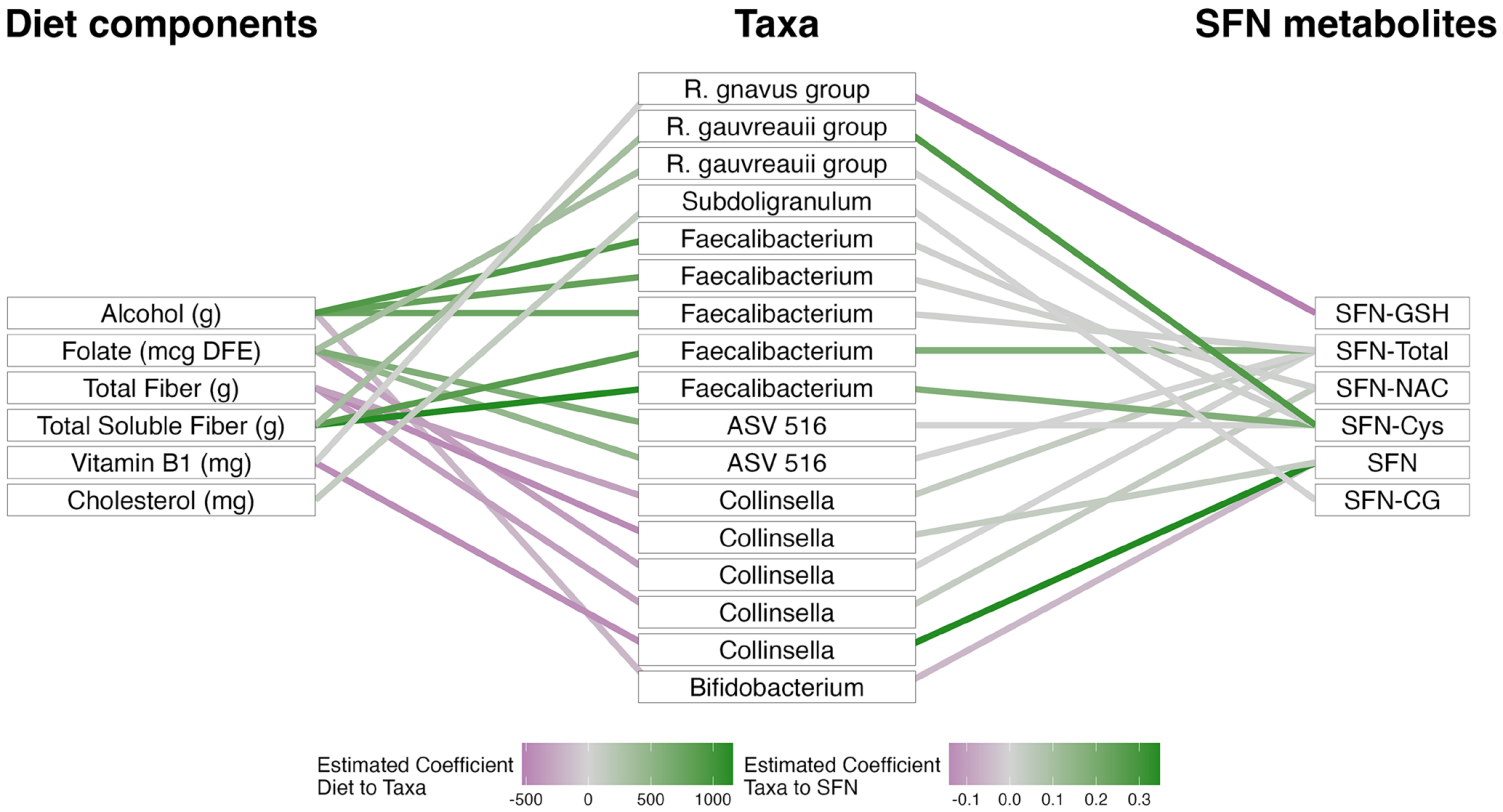
Gut genera that mediate the association between dietary components and SFN metabolites. Dietary components (*X*) are on the left side and connect to mediating genera (*M*) in the middle, which connect to response SFN metabolites (*Y*) on the right side. Color represents the estimated coefficient between the variables. Since the range of coefficients was vastly different between each set of variables, there are two individual scales for each set of relationships, listed below the corresponding set of lines. A mediating genus may appear on multiple connecting lines because it mediates more than one distinct diet to SFN‐metabolite relationship; each line represents a separate mediation model (one dietary component, one mediating genus, and one SFN metabolite), with all distinct models and their FDR‐corrected *p*‐values reported in Table S3. The unclassified *Clostridiales* taxon is denoted ASV516 (*Clostridia* vadinBB60 group; f_NA_ASV516 in the Table S1).

Building on studies that independently linked diet and gut microbiome to SFN metabolism, we analyzed these variables together within an integrated mediation framework (Figure 1B). The abundance of seven bacterial genera mediated the relationship between specific dietary components and SFN metabolites (Figure 2, Table S3): *Collinsella*, *Faecalibacterium*, *Bifidobacterium*, *Subdoligranulum*, an unclassified *Clostridiales* taxon (ASV516), and two *Ruminococcus* groups. Dietary factors such as fiber, B vitamins, alcohol, and cholesterol were associated with SFN metabolites; notably, these relationships were limited to bioactive forms of SFN, with no significant associations observed for the biologically inert metabolite SFN‐NIT.

For example, *Collinsella* abundance was negatively associated with total fiber intake, while positively associated with SFN, SFN‐NAC, and total SFN (Figure 2, Table S3). This pattern suggests that total fiber consumption alters *Collinsella* abundance in ways that ultimately influence the production of SFN and SFN‐NAC (which are likely driving the total SFN association). Given that our mediation analysis was moderately powered, we sought biological validation by testing whether these mediating taxa possessed genes capable of myrosinase‐like activity. To do so, we matched their 16S sequences to complete genomes and searched for myrosinase‐like genes. Several genomes, particularly those from *Collinsella* species, contained such genes (Table S4). Although this approach does not confirm transcriptional activity or rule out undiscovered glucosinolate‐processing enzymes, it supports the hypothesis that these taxa contribute to SFN metabolism. Further work using cultured isolates or single‐cell genomics will be necessary to verify these functional capacities.

### 3.2 Distinct Microbiome Community Subtypes Reflect Host and Dietary Factors

**FIGURE 3:**
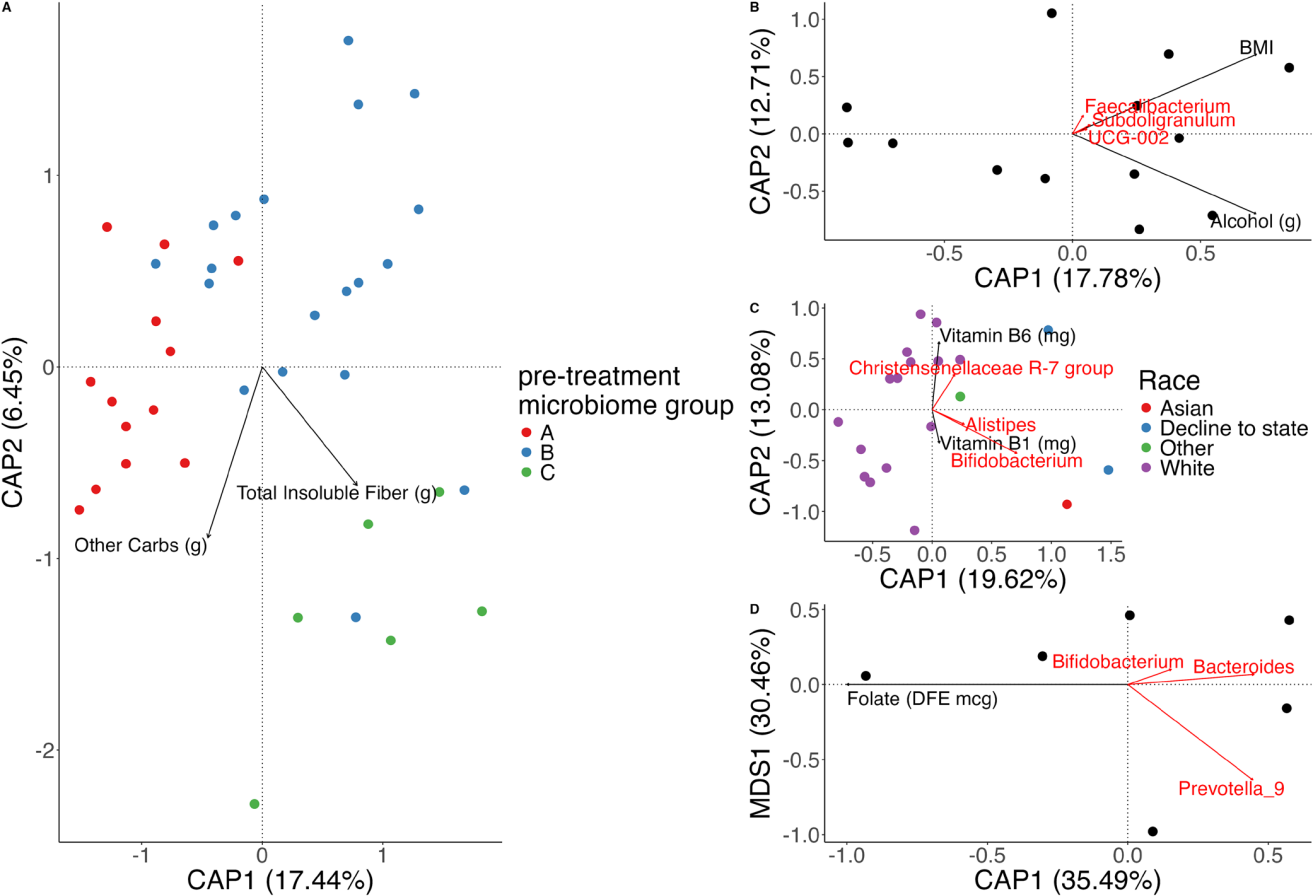
Significant dietary factors describing gut microbiome dissimilarity between participants. Each ordination shown is a dbRDA showing the Bray–Curtis dissimilarity (a beta‐diversity metric) explained by an optimized model using the predictor variables shown in each panel. CAP1 or MDS1 and CAP2 axes are Euclidean distances represent how much variation in the gut microbial samples are explained by the graph, with percent of microbiome variation explained listed on each graph axis in the parentheses. (A) shows metadata variables that best describe the beta‐diversity the whole dataset with all participants across all microbiome groups. Microbiome group is represented by color, and vectors overlayed on the ordination show how levels of “other carbohydrates” (i.e., organic acids, pentose sugars, sugar alcohols, starches, and gums) and “total insoluble fiber” are associated with gut microbiome dissimilarity among participants. (B–D) represent the plots of significant variables from Table 1 for group A (panel B), group B (panel C), and group C (panel D). Colors in panel C represent race. Black vectors represent the association between levels of dietary components from Table 1 and gut microbiome differences in ordination space. Red vectors represent the top 3 taxonomic abundances that most strongly correlate with gut microbiome differences in each ordination.

During initial data exploration, we observed that participants' gut microbiomes clustered into distinct groups prior to broccoli sprout consumption, independent of any experimental manipulation. Cluster analysis revealed three gut microbiome subtypes within the study population (Figure 3A), each distinguished by characteristic taxonomic composition. Consistent with prior enterotype studies, group A exhibited the highest proportion of *Bacteroides*, group B hosted reduced *Bacteroides* with increased *Ruminococcus*, and group C was enriched in *Prevotella* (Figure S1) (Arumugam et al. 2011; Wu et al. 2011). We found that microbiome group membership was best explained by age, race, weight, and consumption levels of alcohol, total soluble fiber, disaccharides, and manganese (Gaussian linear regression; alpha = 0.05; Table S5). Differential abundance testing further supported these compositional distinctions: ANCOM identified *Bacteroides* and the Christensenellaceae R‐7 group as differentially abundant between groups A and B (Figure S2). The greater *Bacteroides* abundance in group A is consistent with the enterotype‐like structure described above (Figure S1), providing further support that these groups have compositional differences. We then examined which dietary variables were associated with gut microbiome beta‐diversity across all participants and within each subtype to identify distinct drivers of community variation. The dietary components shaping overall microbiome dissimilarity (Figure 3A) differed from those that structured diversity within individual subtypes (Figure 3B–D). Across the full cohort, total insoluble fiber (g) and “other” carbohydrates (total carbohydrates – [total fiber + total mono‐ and di‐saccharides]; includes organic acids, pentose sugars, sugar alcohols, starches, and gums) were the strongest predictors of beta‐diversity (Figure 3A, Table 1). In contrast, different dietary and host factors explained variation within each subtype: alcohol intake and BMI for group A (Figure 3B), race and vitamins B6 and B1 for group B (Figure 3C), and folate intake for group C (Figure 3D; Table 1). These patterns in ordination space were accompanied by distinct taxonomic abundance changes (red vectors in Figure 3B–D), indicating which microbial abundances vary along the same environmental and dietary axes. Together, this shows that each microbiome subtype is associated with a unique set of covariate‐taxon relationships that structure beta‐diversity.

**TABLE 1:** Statistical results showing which covariates explain gut microbiome pre‐treatment group membership. PERMANOVA was used to calculate significance based on the optimal regression model (*p*‐values FDR‐corrected; only significant variables shown). “All groups” refers to the full dataset, including groups A, B, and C together, that is, variables explaining the gut microbiome beta‐diversity across the whole study. Gut microbiome beta‐diversity in groups A, B, and C were subsequently modeled independently by the same predictors, listed below in the table.

Gut microbiome beta‐diversity data used as response variable	Predictor variables	*R* ^2^	*p* (alpha = 0.05)
All groups	Total insoluble fiber (g)	0.06959	0.008
All groups	“Other” carbohydrates (total carbohydrates – [total fiber + total mono‐ and di‐saccharides]; that is, organic acids, pentose sugars, sugar alcohols, starches, and gums) (g)	0.06225	0.008
Group A	Alcohol (g)	0.15180	0.01
Group A	BMI	0.15312	0.01
Group B	Race	0.28233	0.01
Group B	Vitamin B6 (mg)	0.09252	0.01
Group B	Vitamin B1 (mg)	0.08570	0.02
Group C	Folate (DFE mcg)	0.35491	0.02

### 3.3 Prediction 2: Pre‐Intervention Diet Mediates the Relationship Between Gut Microbiome Group and SFN Metabolites

**FIGURE 4:**
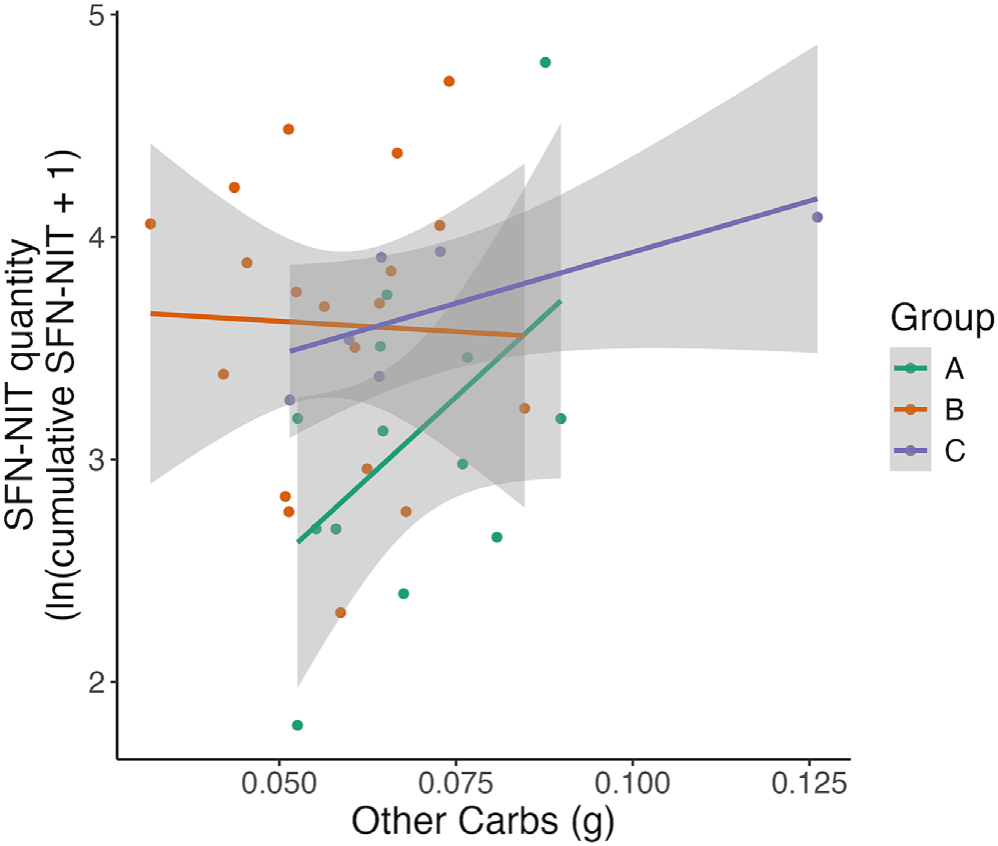
Graph showing the relationships in the significant mediation result of prediction 2 (SFN‐NIT ~ other carbohydrates). Natural log cumulative SFN‐nitrile in urine over time versus “other carbohydrate” consumption in grams is shown, with colored points representing the data and colored lines representing the linear model trends for each microbiome group. The gray shadow around each line is the 95% confidence interval for each line.

Prior research shows that diet influences both gut microbiome composition and SFN metabolism (Carmody et al. 2024; David et al. 2014; Ley et al. 2008; Li et al. 2009; Muegge et al. 2011; Bouranis, Choi, et al. 2021). Building on this evidence, and our findings from prediction 1 and the distinct microbiome subtypes identified above, we tested whether pre‐intervention diet mediated the relationship between microbiome group membership and SFN metabolites. Mediation analysis found that consumption of “other” carbohydrates, which includes organic acids, pentose sugars, sugar alcohols, starches, and gums, was associated with gut microbiome group membership, which in turn was associated with variation in urinary SFN‐NIT (FDR‐corrected *p* = 0.148; Figure 4). This *p*‐value does not meet conventional significance thresholds and should be interpreted as a suggestive, hypothesis‐generating association. Unlike prediction 1, where mediation involved only bioactive forms of SFN, this pathway implicated SFN‐NIT, the biologically inert form of SFN. Participants with A‐ and C‐type microbiomes exhibited a positive association between “other” carbohydrates and SFN‐NIT, whereas those with B‐type microbiomes showed the opposite trend (Figure 4). These results suggest that individuals with A‐ and C‐type microbiomes (*Bacteroides* and *Prevotella* dominated, respectively) may harbor microbial communities that favor conversion of glucosinolates into inert SFN‐NIT, particularly when consuming higher amounts of these types of carbohydrates, whereas the B‐type microbiome may favor alternative, bioactive pathways. Future experiments should endeavor to confirm these associations in vitro and clarify whether different microbiome types differ in their SFN metabolizing abilities.

**FIGURE 5:**
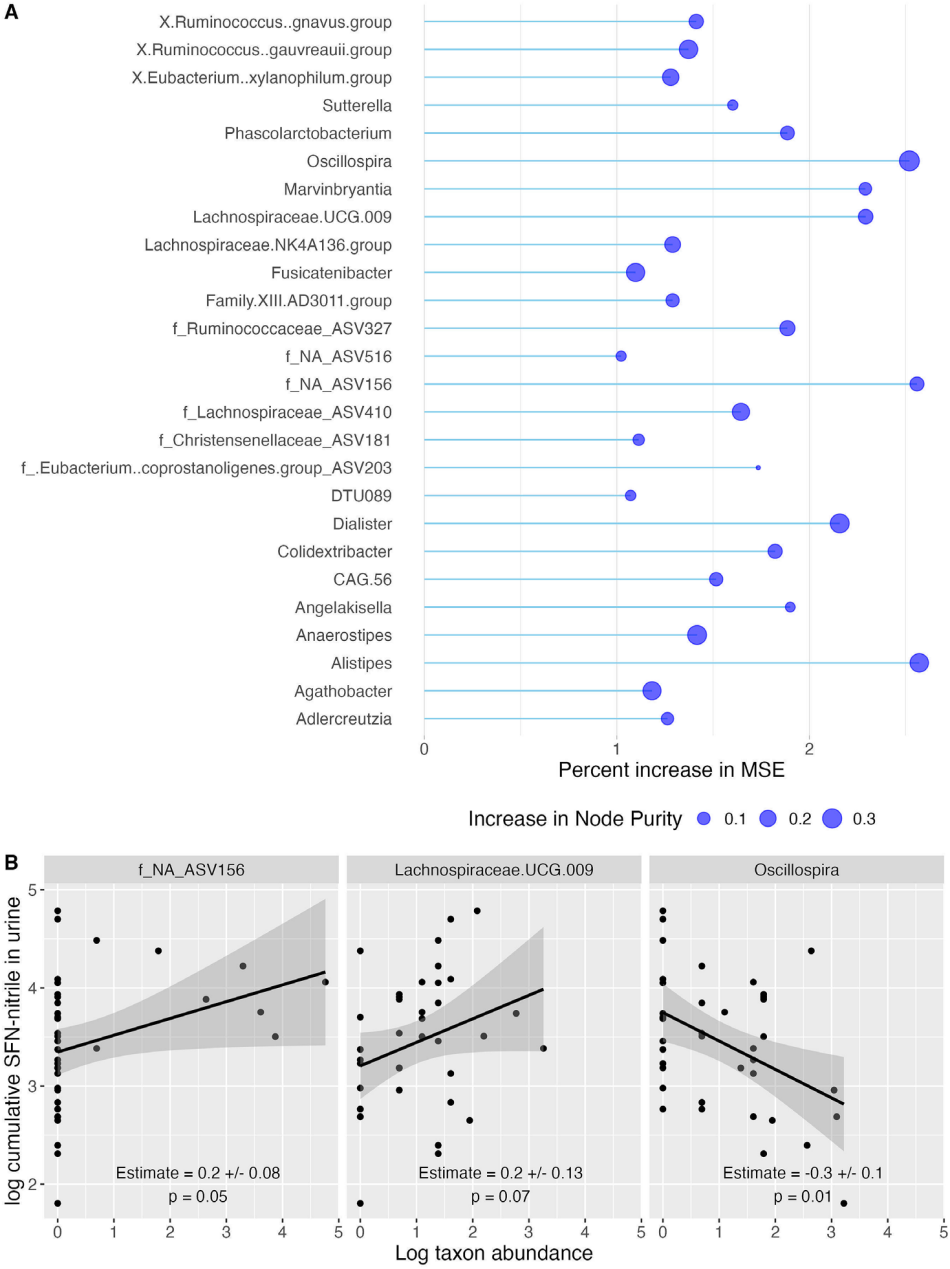
Taxa explaining urinary SFN‐NIT levels. (A) Important taxa determining SFN‐NIT levels in urine based on a random forest regression. Percent increase in *MSE* and increase in node purity are proxies for importance of a taxon's abundance in explaining SFN‐NIT measurements. Percent increase in *MSE* is shown on the *x*‐axis. Taxa with a percent increase in *MSE* > 1 are shown on the *y*‐axis. The size of the circle indicates the increase in node purity that occurs when the taxon is used to model SFN‐NIT; the larger the circle, the more important a taxon is in predicting SFN‐NIT. (B) The relationship between taxon abundance and SFN‐NIT production in the top 3 most important taxa. The *x*‐axis shows the taxon abundance of the top 3 taxa associated significantly with SFN‐NIT (Random forest regression: Percent increase in *MSE* > 2, percent increase in node purity > 0.1 and Gaussian linear regression: Top 3 lowest *p*‐values shown). The *y*‐axis shows the SFN‐NIT measures in urine. The line represents the line of best fit from the Gaussian linear regression, with the 95% confidence interval shown in gray. Slope estimate and *p*‐value from this model are listed at the bottom of each graph.

Because SFN‐NIT concentrations were linked to overall composition, we next examined whether specific taxa linked to SFN‐NIT, representing putatively associated taxa to target for future experimental efforts. Random forest regression, used as a feature‐filtering step to rank taxa by importance to SFN‐NIT, identified 26 taxa that associated with SFN‐NIT across participants (percent increase in mean squared error [MSE] per taxon > 1; Figure 5A). These included well‐characterized gut clades such as *Ruminococcus*, Christensenellaceae, and Lachnospiraceae (Atwell et al. 2015; Ross et al. 2024; Crost et al. 2023; Vacca et al. 2020). We confirmed the association between these putatively influential taxa and SFN‐NIT measures using Gaussian linear regression (top 3 most significant associations shown in Figure 5B). These findings identify specific bacterial taxa that merit further culturing and genomic investigation to confirm their capacity for SFN‐NIT production and elucidate the enzymatic mechanisms involved.

## 4 Discussion

Understanding how diet and the gut microbiota influence SFN metabolism is important in understanding differences in inter‐individual responses and improving the design and personalization of cruciferous vegetable interventions. Previous studies have typically examined these factors in isolation (Li et al. 2009; Bouranis et al. 2024, 2023, 2022; Bouranis, Choi, et al. 2021; Ross et al. 2024), whereas our work integrates all three (diet, gut microbiota, and SFN metabolites) using mediation modeling, a causal inference method. This approach allowed us to test two primary predictions: (1) whether gut microbes mediate the relationship between pre‐intervention diet and SFN metabolism, and (2) whether diet mediates the relationship between microbiome composition and SFN metabolism. Our results support the hypothesis that habitual diet shapes the gut microbiome in ways that influence individual responses to broccoli sprout consumption. The interaction between diet and gut microbiome composition likely explains, at least in part, the interindividual variation previously reported in prior broccoli sprout feeding trials (Bouranis et al. 2024; Bouranis, Choi, et al. 2021), though other physiological and environmental factors likely also contribute. This individualized diet‐microbiome‐SFN relationship provides insight into how routine dietary patterns may modulate the efficacy of cruciferous vegetable interventions. Food intake acts as a resource for gut microbial communities; different taxa possess distinct metabolic capacities to degrade food components, influencing their growth and the metabolic products of digestion (Ross et al. 2024; Ramos and Martín 2021; Zhang 2022). If certain taxa are absent, individuals may lack the metabolic potential to convert specific dietary compounds, such as glucosinolates from cruciferous vegetables, into bioactive SFN metabolites.

We identified evidence in support of two complementary mechanisms linking diet, gut microbiota, and SFN response. First, in line with prediction 1, the abundance of seven bacterial gut genera mediated the effect of six different dietary components on bioactive SFN metabolite levels. Second, in line with prediction 2, participants clustered into three baseline gut microbiome subtypes that exhibited different SFN‐NIT responses depending on the consumption of certain carbohydrate classes. Collectively, these findings demonstrate that SFN metabolism depends not only on dietary composition but also on the underlying microbiome structure that modulates how dietary inputs are processed. At the community level, three distinct microbiome types produced divergent metabolic responses; at the taxon level, differences in the abundance of mediating taxa, such as *Collinsella*, *Bifidobacterium*, and *Ruminococcus* species, dictate whether pre‐intervention diet favored the production of bioactive or inert SFN forms.

The abundance of several bacterial genera mediated the relationship between pre‐intervention diet and SFN metabolites following broccoli sprout consumption. These genera included *Collinsella*, *Faecalibacterium*, an unclassified Clostridiales taxon, *Bifidobacterium*, two *Ruminococcus* groups, and *Subdoligranulum*. Many of these taxa have previously been associated with host metabolic health. For example, *Subdoligranulum* abundance is positively correlated with HDL cholesterol and negatively correlated with adiposity, insulin resistance, levels of leptin, and inflammatory bowel disease (IBD) and IBD‐related metabolites (Lloyd‐Price et al. 2019; Van Hul et al. 2020). Moreover, Bouranis et al. found that *Subdoligranulum* increased in abundance when human fecal samples were incubated with cruciferous vegetable material (Bouranis et al. 2023). Additionally, *R. gauvreauii* has been shown to degrade dietary fiber in the human gut (Bouranis, Beaver, and Ho 2021), and in our study, higher total soluble fiber intake was associated with increased *R. gauvreauii* abundance and higher SFN‐Cys levels (Figure 2, Table 1).

Similarly, *Collinsella* abundance decreased with higher total fiber, and its elevated abundance was associated with higher SFN, SFN‐NAC, and the total of all SFN metabolites, consistent with previous observations linking *Collinsella* to low fiber diets (Gomez‐Arango et al. 2018). *Collinsella* mediated the relationships between B vitamin intake and SFN production: folate and vitamin B1 intake reduced *Collinsella* abundance, whereas greater *Collinsella* abundance increased both “total SFN” and “SFN” metrics in the model (Figure 2, Table 1). *Collinsella aerofaciens* , in particular, produces vitamin B6 (Hossain et al. 2022; Magnúsdóttir et al. 2015) and has been linked to inflammation and obesity (Gomez‐Arango et al. 2018; Astbury et al. 2020).

These taxa were associated with multiple dietary components (fiber, B vitamins, alcohol, and cholesterol) that collectively shaped bioactive SFN metabolite levels. Genome analysis further revealed that representative strains from these genera encode putative myrosinase‐like enzymes, with *Collinsella* genomes exhibiting the greatest diversity of such genes. While genomic inference alone cannot confirm enzyme expression or activity, these results highlight candidate taxa for in vitro validation of SFN‐metabolizing capacity. Together, these findings suggest that a subset of gut bacterial genera mediate how diet influences SFN bioavailability and thus the physiological response to broccoli sprout consumption.

We also identified three baseline gut microbiota subtypes that explained variation in SFN‐NIT responses, mediated by the consumption of certain non‐fiber carbohydrates. SFN and most of its metabolites are bioactive and confer health benefits, whereas SFN‐NIT is biologically inactive (Bouranis et al. 2024; Bouranis, Beaver, and Ho 2021; Basten et al. 2002). Each microbiome subtype was associated with distinct participant characteristics, such as BMI and race, as well as dietary patterns. These findings suggest that both host attributes and diet shape the gut microbiome into compositional subtypes that, in turn, predict metabolic outcomes following broccoli sprout consumption. This result aligns with previous work that links BMI and body mass to SFN metabolism (Charron et al. 2018; Fahey et al. 2019). The existence of distinct gut microbiome subtypes may therefore help to explain the interindividual variation consistently reported in cruciferous vegetable feeding trials (Bouranis et al. 2024; Bouranis, Choi, et al. 2021; Jamieson et al. 2024).

In the future, microbiome typing could enable personalized interventions, such as tailored diets, probiotics, or synbiotics, designed to enrich beneficial taxa and enhance SFN bioactivity. However, this approach will require deeper characterization of the gut microbial functions and their metabolic roles under different dietary contexts. Numerous factors beyond diet and host characteristics influence microbiome structure, including drug use (Jernberg et al. 2010; Weersma et al. 2020), geography (Gupta et al. 2017), chemical exposures (Claus et al. 2016), pregnancy (Isolauri et al. 2017), and physical activity (Mailing et al. 2019). Nevertheless, diet remains a dominant driver of gut microbial composition and an important determinant of SFN metabolism (Li et al. 2009, 2011; Liu et al. 2017; Bouranis, Choi, et al. 2021).

Overall, our findings support a complex interplay between diet, microbiome composition, and SFN metabolism that collectively determines whether bioactive metabolites are produced, and thus the degree to which individuals may benefit from broccoli sprout consumption. These complex interactions warrant further investigation to disentangle the respective roles of human (e.g., mastication) and microbial processes in SFN metabolism. Clarifying these mechanisms will require targeted culturing and metabolic testing of specific gut taxa identified in this study. The extent to which gut microbes produce SFN metabolites remains incompletely understood, in part because human and bacterial activities are difficult to separate experimentally. Before broccoli‐derived glucosinolates reach the gut, mastication and plant myrosinase activity convert them into various unstable intermediates, only some of which lead to bioactive SFN (Bouranis, Beaver, and Ho 2021). Once in the gut, microbial metabolism of glucoraphanin by myrosinase‐like enzymes further transforms these intermediates into SFN and or its derivatives (Bouranis, Choi, et al. 2021). In both cases, a portion of glucosinolates are converted into biologically inert nitriles (Bouranis et al. 2024; Bouranis, Choi, et al. 2021; Basten et al. 2002). Our prior research found that taxa such as Clostridiaceae are associated with a high nitrile abundance, whereas Ruminococcaceae, Enterobacteriaceae, and Bacteroidales are associated with lower nitrile levels (Bouranis et al. 2024). Our findings support these observations, identifying similar groups of bacteria as mediators of dietary effects on SFN nitrile vs. non‐nitrile formation. It is therefore possible that the presence or absence of specific taxa determines the types of SFN metabolites produced upon consuming broccoli. In particular, *Ruminococcus*, *Collinsella*, *Subdoligranulum*, and *Dialister*, among others identified in this study, represent promising candidates for targeted culturing and in vitro characterization of SFN‐metabolizing potential.

Taken together, our results underscore the importance of the gut microbiota as a key mediator between diet and health, shaping the production of bioactive compounds from food. Variation in gut microbial community types contributes, at least in part, to the high interindividual variation observed in broccoli sprout interventions (Bouranis et al. 2024; Bouranis, Choi, et al. 2021). This work does not delve into the mechanism behind these patterns, but points to future candidate taxa that should next be tested in vitro. Identifying the microbial and dietary factors underlying this variation supports the advancement of precision nutrition approaches, in which interventions are tailored to an individual's gut microbiome profile. Specifically, optimizing bioactive SFN metabolite production through personalized dietary or probiotic strategies could enhance the preventative and therapeutic benefits of cruciferous vegetable consumption.

## Author Contributions

**Laura M. Beaver:** writing – review and editing, data curation, methodology. **Alexandra Alexiev:** writing – original draft, writing – review and editing, methodology, visualization, formal analysis, investigation. **Emily Ho:** conceptualization, investigation, funding acquisition, writing – review and editing, project administration, supervision, resources. **Jan F. Stevens:** conceptualization, investigation, funding acquisition, writing – review and editing, supervision, project administration, resources. **John A. Bouranis:** validation, methodology, writing – review and editing, data curation, investigation. **Carmen P. Wong:** methodology, investigation, writing – review and editing, supervision. **Thomas J. Sharpton:** conceptualization, investigation, funding acquisition, writing – review and editing, project administration, supervision, resources.

## Funding

This work was supported by the National Institute of Food and Agriculture, NIFA‐2020‐67001‐31214; Oregon Agricultural Experimental Station, W5002, OR00735; National Institutes of Health, P30ES030287; National Institute of Environmental Health Sciences, T32ES007060.

## Conflicts of Interest

The authors declare no conflicts of interest.

## Supporting information


**Figure S1:** Enterotypes describe gut microbiome subtypes to an extent in the present study. A stacked bar chart shows taxa indicative of enterotypes in green, orange, and purple (those in the gray other category being taxa not indicative of enterotypes). The *y*‐axis shows taxonomic proportions in each microbiome subtype grouping, A, B, or C, in the dataset.
**Figure S2:** Differentially abundant genera between microbiome groups A and B identified by ANCOM. Group C was excluded due to low sample size. Bars show the relative abundance of the two genera detected as differentially abundant (Bacteroides and the Christensenellaceae R‐7 group) under conservative ANCOM thresholds (detection cutoff 0.7 with significant effect size), colored by pre‐treatment microbiome group. Relative abundances are shown for ease of visualization of the ANCOM‐detected taxa. The test itself operates on log‐ratio‐transformed abundances.
**Table S1:** Significant associations between dietary components (X) and SFN metabolites (Y), which subsequently informed the mediation model for prediction 1.
**Table S2:** Results for mediation analysis using ASV genus abundances as explanatory variables (X), dietary components as mediators (M), and SFN metabolites in urine as responses (Y). ACME *p*‐values are shown and are FDR‐corrected (alpha = 0.1).
**Table S3:** Prediction 1 mediation results. Gut genera that mediate the association between dietary components and SFN metabolites. Mediation (ACME) *p*‐values were FDR‐corrected (alpha = 0.2).
**Table S4:** mediating taxa from Prediction 1 mediation results that matched genomes containing myrosinase‐like genes. We checked to see if these mediating genera matched to already sequenced genomes that contain enzymes that break down glucoraphanin, which are myrosinase‐like genes in bacteria.
**Table S5:** Gaussian linear regression model results from testing which covariates significantly describe gut microbiome subtype groupings. *p*‐values (alpha = 0.05) show an asterisk for significant and period for nearly significant values.

## Data Availability

Sequences are hosted on NCBI's SRA database under BioProject PRJNA953404. The code is posted to github at https://github.com/aalexiev/Broccoli_MBmediation.

## References

[fsn372211-bib-0001] Albaser, A. , E. Kazana , M. H. Bennett , et al. 2016. “Discovery of a Bacterial Glycoside Hydrolase Family 3 (GH3) β‐Glucosidase With Myrosinase Activity From a Citrobacter Strain Isolated From Soil.” Journal of Agricultural and Food Chemistry 64: 1520–1527. 26820976 10.1021/acs.jafc.5b05381

[fsn372211-bib-0002] Arumugam, M. , J. Raes , E. Pelletier , et al. 2011. “Enterotypes of the Human Gut Microbiome.” Nature 473: 174–180. 21508958 10.1038/nature09944 PMC3728647

[fsn372211-bib-0003] Astbury, S. , E. Atallah , A. Vijay , G. P. Aithal , J. I. Grove , and A. M. Valdes . 2020. “Lower Gut Microbiome Diversity and Higher Abundance of Proinflammatory Genus Collinsellaare Associated With Biopsy‐Proven Nonalcoholic Steatohepatitis.” Gut Microbes 11: 569–580. 31696774 10.1080/19490976.2019.1681861 PMC7524262

[fsn372211-bib-0004] Atwell, L. L. , A. Hsu , C. P. Wong , et al. 2015. “Absorption and Chemopreventive Targets of Sulforaphane in Humans Following Consumption of Broccoli Sprouts or a Myrosinase‐Treated Broccoli Sprout Extract.” Molecular Nutrition & Food Research 59: 424–433. 25522265 10.1002/mnfr.201400674 PMC4394840

[fsn372211-bib-0005] Azarenko, O. , M. A. Jordan , and L. Wilson . 2014. “Erucin, the Major Isothiocyanate in Arugula (*Eruca sativa*), Inhibits Proliferation of MCF7 Tumor Cells by Suppressing Microtubule Dynamics.” PLoS One 9: e100599. 24950293 10.1371/journal.pone.0100599 PMC4065051

[fsn372211-bib-0006] Basten, G. P. , Y. Bao , and G. Williamson . 2002. “Sulforaphane and Its Glutathione Conjugate but Not Sulforaphane Nitrile Induce UDP‐Glucuronosyl Transferase (UGT1A1) and Glutathione Transferase (GSTA1) in Cultured Cells.” Carcinogenesis 23: 1399–1404. 12151360 10.1093/carcin/23.8.1399

[fsn372211-bib-0007] Beaver, L. M. , C. V. Lohr , J. D. Clarke , et al. 2018. “Broccoli Sprouts Delay Prostate Cancer Formation and Decrease Prostate Cancer Severity With a Concurrent Decrease in HDAC3 Protein Expression in Transgenic Adenocarcinoma of the Mouse Prostate (TRAMP) Mice.” Current Developments in Nutrition 2: nzy002. 30019025 10.1093/cdn/nzy002 PMC6041877

[fsn372211-bib-0008] Bonnesen, C. , I. M. Eggleston , and J. D. Hayes . 2001. “Dietary Indoles and Isothiocyanates That Are Generated From Cruciferous Vegetables Can Both Stimulate Apoptosis and Confer Protection Against DNA Damage in Human Colon Cell Lines.” Cancer Research 61: 6120–6130. 11507062

[fsn372211-bib-0009] Bouranis, J. A. , L. M. Beaver , J. Choi , et al. 2021. “Composition of the Gut Microbiome Influences Production of Sulforaphane‐Nitrile and Iberin‐Nitrile From Glucosinolates in Broccoli Sprouts.” Nutrients 13: 3013. 34578891 10.3390/nu13093013 PMC8468500

[fsn372211-bib-0010] Bouranis, J. A. , L. M. Beaver , and E. Ho . 2021. “Metabolic Fate of Dietary Glucosinolates and Their Metabolites: A Role for the Microbiome.” Frontiers in Nutrition 8: 8. 10.3389/fnut.2021.748433. PMC8492924 34631775

[fsn372211-bib-0011] Bouranis, J. A. , L. M. Beaver , D. Jiang , et al. 2022. “Interplay Between Cruciferous Vegetables and the Gut Microbiome: A Multi‐Omic Approach.” Nutrients 15: 42. 36615700 10.3390/nu15010042 PMC9824405

[fsn372211-bib-0012] Bouranis, J. A. , L. M. Beaver , C. P. Wong , et al. 2024. “Sulforaphane and Sulforaphane‐Nitrile Metabolism in Humans Following Broccoli Sprout Consumption: Inter‐Individual Variation, Association With Gut Microbiome Composition, and Differential Bioactivity.” Molecular Nutrition & Food Research 68: 2300286. 10.1002/mnfr.202300286 PMC10922398 38143283

[fsn372211-bib-0013] Bouranis, J. A. , C. P. Wong , L. M. Beaver , et al. 2023. “Sulforaphane Bioavailability in Healthy Subjects Fed a Single Serving of Fresh Broccoli Microgreens.” Food 12: 3784. 10.3390/foods12203784 PMC10606698 37893677

[fsn372211-bib-0014] Carmody, R. N. , K. Varady , and P. J. Turnbaugh . 2024. “Digesting the Complex Metabolic Effects of Diet on the Host and Microbiome.” Cell 187: 3857–3876. 39059362 10.1016/j.cell.2024.06.032 PMC11309583

[fsn372211-bib-0015] Charron, C. S. , B. T. Vinyard , S. A. Ross , H. E. Seifried , E. H. Jeffery , and J. A. Novotny . 2018. “Absorption and Metabolism of Isothiocyanates Formed From Broccoli Glucosinolates: Effects of BMI and Daily Consumption in a Randomised Clinical Trial.” British Journal of Nutrition 120: 1370–1379. 30499426 10.1017/S0007114518002921

[fsn372211-bib-0016] Clarke, J. D. , A. Hsu , K. Riedl , et al. 2011. “Bioavailability and Inter‐Conversion of Sulforaphane and Erucin in Human Subjects Consuming Broccoli Sprouts or Broccoli Supplement in a Cross‐Over Study Design.” Pharmacological Research 64: 456–463. 21816223 10.1016/j.phrs.2011.07.005 PMC3183106

[fsn372211-bib-0017] Claus, S. P. , H. Guillou , and S. Ellero‐Simatos . 2016. “The Gut Microbiota: A Major Player in the Toxicity of Environmental Pollutants?.” NPJ Biofilms and Microbiomes 2: 1–11. 28721242 10.1038/npjbiofilms.2016.3 PMC5515271

[fsn372211-bib-0018] Cordeiro, R. P. , J. H. Doria , G. G. Zhanel , R. Sparling , and R. A. Holley . 2015. “Role of Glycoside Hydrolase Genes in Sinigrin Degradation by *E. coli* O157:H7.” International Journal of Food Microbiology 205: 105–111. 25897994 10.1016/j.ijfoodmicro.2015.04.008

[fsn372211-bib-0019] Crost, E. H. , E. Coletto , A. Bell , and N. Juge . 2023. “ *Ruminococcus gnavus*: Friend or Foe for Human Health.” FEMS Microbiology Reviews 47: fuad014. 37015876 10.1093/femsre/fuad014 PMC10112845

[fsn372211-bib-0047] Curioni, C. C. , A. C. F. da Silva , A. da Silva Pereira , and M. Mocellin . 2022. “The Role of Dietary Habits on Development and Progress of Risk Factors of Chronic Non‐Communicable Diseases.” In Healthy Lifestyle: From Pediatrics to Geriatrics, edited by R. Kelishadi , 105–129. Springer International Publishing.

[fsn372211-bib-0020] David, L. A. , C. F. Maurice , R. N. Carmody , et al. 2014. “Diet Rapidly and Reproducibly Alters the Human Gut Microbiome.” Nature 505: 559–563. 24336217 10.1038/nature12820 PMC3957428

[fsn372211-bib-0021] Fahey, J. W. , K. L. Wade , K. K. Stephenson , et al. 2019. “Bioavailability of Sulforaphane Following Ingestion of Glucoraphanin‐Rich Broccoli Sprout and Seed Extracts With Active Myrosinase: A Pilot Study of the Effects of Proton Pump Inhibitor Administration.” Nutrients 11: 1489. 31261930 10.3390/nu11071489 PMC6682992

[fsn372211-bib-0022] Fahey, J. W. , S. L. Wehage , W. D. Holtzclaw , et al. 2012. “Protection of Humans by Plant Glucosinolates: Efficiency of Conversion of Glucosinolates to Isothiocyanates by the Gastrointestinal Microflora.” Cancer Prevention Research (Philadelphia, Pa.) 5: 603–611. 22318753 10.1158/1940-6207.CAPR-11-0538 PMC4618677

[fsn372211-bib-0023] Gomez‐Arango, L. F. , H. L. Barrett , S. A. Wilkinson , et al. 2018. “Low Dietary Fiber Intake Increases Collinsella Abundance in the Gut Microbiota of Overweight and Obese Pregnant Women.” Gut Microbes 9: 189–201. 29144833 10.1080/19490976.2017.1406584 PMC6219589

[fsn372211-bib-0024] Gupta, V. K. , S. Paul , and C. Dutta . 2017. “Geography, Ethnicity or Subsistence‐Specific Variations in Human Microbiome Composition and Diversity.” Frontiers in Microbiology 8: 1162. 10.3389/fmicb.2017.01162. 28690602 PMC5481955

[fsn372211-bib-0025] Hammer, A. J. , C. A. Gaulke , M. Garcia‐Jaramillo , et al. 2024. “Gut Microbiota Metabolically Mediate Intestinal Helminth Infection in Zebrafish.” mSystems 9: e0054524. 39191377 10.1128/msystems.00545-24 PMC11406965

[fsn372211-bib-0026] Hossain, K. S. , S. Amarasena , and S. Mayengbam . 2022. “B Vitamins and Their Roles in Gut Health.” Microorganisms 10: 1168. 35744686 10.3390/microorganisms10061168 PMC9227236

[fsn372211-bib-0027] Isolauri, E. , P. M. Sherman , and W. A. Walker . 2017. Intestinal Microbiome: Functional Aspects in Health and Disease: 88th Nestlé Nutrition Institute Workshop, Playa Del Carmen. Karger Medical And Scientific Publishers.

[fsn372211-bib-0028] Jamieson, P. E. , E. B. Smart , J. A. Bouranis , et al. 2024. “Gut Enterotype‐Dependent Modulation of Gut Microbiota and Their Metabolism in Response to Xanthohumol Supplementation in Healthy Adults.” Gut Microbes 16: 2315633. 38358253 10.1080/19490976.2024.2315633 PMC10878022

[fsn372211-bib-0029] Jernberg, C. , S. Löfmark , C. Edlund , and J. K. Jansson . 2010. “Long‐Term Impacts of Antibiotic Exposure on the Human Intestinal Microbiota.” Microbiology (Reading) 156: 3216–3223. 20705661 10.1099/mic.0.040618-0

[fsn372211-bib-0030] Ley, R. E. , M. Hamady , C. Lozupone , et al. 2008. “Evolution of Mammals and Their Gut Microbes.” Science 320: 1647–1651. 18497261 10.1126/science.1155725 PMC2649005

[fsn372211-bib-0031] Li, F. , M. A. J. Hullar , S. A. A. Beresford , and J. W. Lampe . 2011. “Variation of Glucoraphanin Metabolismin Vivoandex Vivoby Human Gut Bacteria.” British Journal of Nutrition 106: 408–416. 21342607 10.1017/S0007114511000274 PMC3137642

[fsn372211-bib-0032] Li, F. , M. A. J. Hullar , Y. Schwarz , and J. W. Lampe . 2009. “Human Gut Bacterial Communities Are Altered by Addition of Cruciferous Vegetables to a Controlled Fruit‐ and Vegetable‐Free Diet.” Journal of Nutrition 139: 1685–1691. 19640972 10.3945/jn.109.108191 PMC2728691

[fsn372211-bib-0033] Liou, C. S. , S. J. Sirk , C. A. C. Diaz , et al. 2020. “A Metabolic Pathway for Activation of Dietary Glucosinolates by a Human Gut Symbiont.” Cell 180: 717–728.e19. 32084341 10.1016/j.cell.2020.01.023 PMC7515767

[fsn372211-bib-0034] Liu, X. , Y. Wang , J. L. Hoeflinger , B. P. Neme , E. H. Jeffery , and M. J. Miller . 2017. “Dietary Broccoli Alters Rat Cecal Microbiota to Improve Glucoraphanin Hydrolysis to Bioactive Isothiocyanates.” Nutrients 9: 262. 28287418 10.3390/nu9030262 PMC5372925

[fsn372211-bib-0035] Lloyd‐Price, J. , C. Arze , A. N. Ananthakrishnan , et al. 2019. “Multi‐Omics of the Gut Microbial Ecosystem in Inflammatory Bowel Diseases.” Nature 569: 655–662. 31142855 10.1038/s41586-019-1237-9 PMC6650278

[fsn372211-bib-0036] Luang‐In, V. , A. A. Albaser , C. Nueno‐Palop , M. H. Bennett , A. Narbad , and J. T. Rossiter . 2016. “Glucosinolate and Desulfo‐Glucosinolate Metabolism by a Selection of Human Gut Bacteria.” Current Microbiology 73: 442–451. 27301252 10.1007/s00284-016-1079-8

[fsn372211-bib-0037] Luang‐In, V. , A. Narbad , C. Nueno‐Palop , R. Mithen , M. Bennett , and J. T. Rossiter . 2014. “The Metabolism of Methylsulfinylalkyl‐ and Methylthioalkyl‐Glucosinolates by a Selection of Human Gut Bacteria.” Molecular Nutrition & Food Research 58: 875–883. 24170324 10.1002/mnfr.201300377

[fsn372211-bib-0038] Magnúsdóttir, S. , D. Ravcheev , V. de Crécy‐Lagard , and I. Thiele . 2015. “Systematic Genome Assessment of B‐Vitamin Biosynthesis Suggests Co‐Operation Among Gut Microbes.” Frontiers in Genetics 6: 6. 10.3389/fgene.2015.00148. 25941533 PMC4403557

[fsn372211-bib-0039] Mailing, L. J. , J. M. Allen , T. W. Buford , C. J. Fields , and J. A. Woods . 2019. “Exercise and the Gut Microbiome: A Review of the Evidence, Potential Mechanisms, and Implications for Human Health.” Exercise and Sport Sciences Reviews 47: 75–85. 30883471 10.1249/JES.0000000000000183

[fsn372211-bib-0040] Mandal, S. , W. Van Treuren , R. A. White , M. Eggesbø , R. Knight , and S. D. Peddada . 2015. “Analysis of Composition of Microbiomes: A Novel Method for Studying Microbial Composition.” Microbial Ecology in Health and Disease 26: 27663. 26028277 10.3402/mehd.v26.27663 PMC4450248

[fsn372211-bib-0041] Muegge, B. D. , J. Kuczynski , D. Knights , et al. 2011. “Diet Drives Convergence in Gut Microbiome Functions Across Mammalian Phylogeny and Within Humans.” Science 332: 970–974. 21596990 10.1126/science.1198719 PMC3303602

[fsn372211-bib-0042] Neuhouser, M. L. 2019. “The Importance of Healthy Dietary Patterns in Chronic Disease Prevention.” Nutrition Research 70: 3–6. 30077352 10.1016/j.nutres.2018.06.002 PMC6328339

[fsn372211-bib-0043] Palop, M. L. , J. P. Smiths , and B. ten Brink . 1995. “Degradation of Sinigrin by *Lactobacillus agilis* Strain R16.” International Journal of Food Microbiology 26: 219–229. 7577359 10.1016/0168-1605(95)00123-2

[fsn372211-bib-0044] Ramos, S. , and M. Á. Martín . 2021. “Impact of Diet on Gut Microbiota.” Current Opinion in Food Science 37: 83–90.

[fsn372211-bib-0045] Ross, F. C. , D. Patangia , G. Grimaud , et al. 2024. “The Interplay Between Diet and the Gut Microbiome: Implications for Health and Disease.” Nature Reviews. Microbiology 22: 671–686. 39009882 10.1038/s41579-024-01068-4

[fsn372211-bib-0046] Rowland, I. , G. Gibson , A. Heinken , et al. 2018. “Gut Microbiota Functions: Metabolism of Nutrients and Other Food Components.” European Journal of Nutrition 57: 1–24. 10.1007/s00394-017-1445-8 PMC5847071 28393285

[fsn372211-bib-0048] Vacca, M. , G. Celano , F. M. Calabrese , P. Portincasa , M. Gobbetti , and M. De Angelis . 2020. “The Controversial Role of Human Gut Lachnospiraceae.” Microorganisms 8: 573. 32326636 10.3390/microorganisms8040573 PMC7232163

[fsn372211-bib-0049] Van Hul, M. , T. Le Roy , E. Prifti , et al. 2020. “From Correlation to Causality: The Case of *Subdoligranulum* .” Gut Microbes 12: 1849998. 33323004 10.1080/19490976.2020.1849998 PMC7744154

[fsn372211-bib-0050] Vernocchi, P. , F. Del Chierico , and L. Putignani . 2020. “Gut Microbiota Metabolism and Interaction With Food Components.” International Journal of Molecular Sciences 21: 3688. 32456257 10.3390/ijms21103688 PMC7279363

[fsn372211-bib-0051] Weersma, R. K. , A. Zhernakova , and J. Fu . 2020. “Interaction Between Drugs and the Gut Microbiome.” Gut 69: 1510–1519. 32409589 10.1136/gutjnl-2019-320204 PMC7398478

[fsn372211-bib-0052] Wu, G. D. , J. Chen , C. Hoffmann , et al. 2011. “Linking Long‐Term Dietary Patterns With Gut Microbial Enterotypes.” Science 334: 105–108. 21885731 10.1126/science.1208344 PMC3368382

[fsn372211-bib-0053] Zhang, P. 2022. “Influence of Foods and Nutrition on the Gut Microbiome and Implications for Intestinal Health.” International Journal of Molecular Sciences 23: 9588. 36076980 10.3390/ijms23179588 PMC9455721

