# Supplementary material for: The Gut Microbiome and Diet Interact to Dictate Personalized Response to Broccoli Sprout Consumption
Source: Food Sci Nutr. 2026 Aug 5;14(8):e72211. doi: 10.1002/fsn3.72211 (PMC13443138; doi:10.1002/fsn3.72211)
Supplement: Supplementary file 1 [file FSN3-14-e72211-s001.zip › fsn372211-sup-0003-TableS1-S5-FigureS1-S2@Supplementary_Materials.docx]

**Supporting Information**

Table S1: Significant associations between dietary components (X) and SFN metabolites (Y), which subsequently informed the mediation model for prediction 1.

| **SFN type** | **Diet component term** | **p-value (alpha = 0.05)** |
| --- | --- | --- |
| SFN | Alcohol (g) | 0.04057789 |
| SFN | Alcohol^2^ | 0.04121702 |
| SFN | Vitamin B1 (mg) | 0.01150623 |
| SFN | Vitamin B1^2^ | 0.00864462 |
| SFN | Vitamin B1^3^ | 0.00693176 |
| SFN | Vitamin B1^4^ | 0.00624044 |
| SFN | Iron (mg) | 0.01385415 |
| SFN | Iron^2^ | 0.01567245 |
| SFN | Iron^3^ | 0.01811041 |
| SFN | Iron^4^ | 0.02103203 |
| Total SFN | Alcohol (g) | 0.00388453 |
| Total SFN | Alcohol^2^ | 0.01047977 |
| Total SFN | Alcohol^3^ | 0.04411337 |
| Total SFN | Folate (mcg) | 0.03803778 |
| Total SFN | Folate^2^ | 0.03525093 |
| Total SFN | Folate^3^ | 0.03725774 |
| Total SFN | Folate^4^ | 0.04123304 |

Table S2: Results for mediation analysis using ASV genus abundances as explanatory variables (X), dietary components as mediators (M), and SFN metabolites in urine as responses (Y). ACME p-values are shown and are FDR-corrected (alpha = 0.1).

| **Genus (X)** | **Dietary component (M)** | **SFN metabolite (Y)** | **ACME p-value (alpha = 0.1)** |
| --- | --- | --- | --- |
| X.Eubacterium..ruminantium.group | Disaccharides (g) | SFN | 0.04 |
| f_Lachnospiraceae_ASV558 | Disaccharides (g) | SFN | 0.096 |
| X.Eubacterium..ruminantium.group | Folate (mcg) | SFN | 0.084 |
| f_Ruminococcaceae_ASV610 | Copper (mg) | SFN | 0.088 |
| f_Ruminococcaceae_ASV610 | Iron (mg) | SFN | 0.076 |
| Colidextribacter | Monosaccharides (g) | SFN_NIT | 0.068 |
| Lachnospiraceae.FCS020.group | Folate (mcg) | SFN_NIT | 0.092 |
| Alistipes | “Other” carbohydrates (g) | SFN_NIT | 0.092 |
| Bacteroides | “Other” carbohydrates (g) | SFN_NIT | 0.1 |
| Lachnospiraceae.UCG.004 | “Other” carbohydrates (g) | SFN_NIT | 0.072 |
| Adlercreutzia | Iron (mg) | SFN_NIT | 0.076 |
| f_Lachnospiraceae_ASV297 | Iron (mg) | SFN_NIT | 0.1 |
| f_NA_ASV156 | Iron (mg) | SFN_NIT | 0.088 |

Table S3: Prediction 1 mediation results. Gut genera that mediate the association between dietary components and SFN metabolites. Mediation (ACME) p-values were FDR-corrected (alpha = 0.2).

| **Diet** | **Genus** | **SFN** | **Mediation**  **p-value (alpha = 0.2)** |
| --- | --- | --- | --- |
| Cholesterol (mg) | Subdoligranulum | SFN_CG | 0.044 |
| Vitamin B1 (mg) | Collinsella | SFN | 0.056 |
| Total soluble fiber (g) | Faecalibacterium | SFN_Cys | 0.064 |
| Total fiber (g) | Collinsella | SFN_NAC | 0.072 |
| Total soluble fiber (g) | Faecalibacterium | SFN_Tot | 0.08 |
| Folate (mcg) | f_NA_ASV516 (Clostridia vadinBB60 group) | SFN_Tot | 0.084 |
| Folate (mcg) | Collinsella | SFN_Tot | 0.104 |
| Total fiber (g) | Collinsella | SFN | 0.124 |
| Folate (mcg) | X.Ruminococcus..gauvreauii.group | SFN_Cys | 0.128 |
| Total fiber (g) | Collinsella | SFN_Tot | 0.144 |
| Alcohol (g) | Faecalibacterium | SFN_Tot | 0.152 |
| Folate (mcg) | f_NA_ASV516 (Clostridia vadinBB60 group) | SFN_Cys | 0.16 |
| Alcohol (g) | Faecalibacterium | SFN_NAC | 0.164 |
| Vitamin B1 (mg) | X.Ruminococcus..gnavus.group | SFN_GSH | 0.164 |
| Alcohol (g) | Faecalibacterium | SFN_Cys | 0.168 |
| Alcohol (g) | Bifidobacterium | SFN | 0.2 |
| Total soluble fiber (g) | X.Ruminococcus..gauvreauii.group | SFN_Cys | 0.204 |

Table S4: mediating taxa from Prediction 1 mediation results that matched genomes containing myrosinase-like genes. We checked to see if these mediating genera matched to already sequenced genomes that contain enzymes that break down glucoraphanin, which are myrosinase-like genes in bacteria.

| **Taxon** | **Protein-coding genes found** |
| --- | --- |
| [Ruminococcus] gnavus group | 6-phospho-beta-glucosidase (1 copy) |
| Bifidobacterium NA | Beta-glucosidase (1 copy), sulfatase-like hydrolase/transferase (1 copy) |
| Bifidobacterium bifidum | 6-phospho-beta-glucosidase (1 copy) |
| Collinsella NA | 6-phospho-beta-glucosidase (3 copies), sulfatase-like hydrolase/transferase (2 copies), arylsulfatase (2 copies), sulfatase (1 copy) |
| Collinsella aerofaciens | 6-phospho-beta-glucosidase (2 copies), beta-glucosidase, blgX (1 copy), sulfatase-like hydrolase/transferase (1 copy) |
| Collinsella bouchesdurhonensis | none |
| Collinsella tanakaei | 6-phospho-beta-glucosidase (2 copies), anaerobic sulfatase maturase (1 copy), arylsulfatase (1 copy), sulfatase (1 copy) |
| Faecalibacterium NA | sulfatase-like hydrolase/transferase (1 copy), alkyl/aryl-sulfatase (1 copy) |
| Faecalibacterium prausnitzii | 6-phospho-beta-glucosidase (1 copy), GH1 family beta-glucosidase (1 copy) |
| Clostridia NA (really poor match) | anaerobic sulfatase maturase (1 copy), sulfatase-like hydrolase/transferase (1 copy) |
| Subdoligranulum NA | GH1 family beta-glucosidase (1 copy), beta-glucosidase (7 copies), arylsulfatase (1 copy), anaerobic sulfatase maturase (1 copy), mucin desulfatase (2 copies) |
| Subdoligranulum variabile | 6-phospho-beta-glucosidase (2 copies), anaerobic sulfatase maturase (1 copy), sulfatase-like hydrolase/transferase (1 copy) |
| [Ruminococcus] gauvreauii group | 6-phospho-beta-glucosidase (1 copy), beta-glucosidase bglX (1 copy), beta-glucosidase (3 copies), sulfatase-like hydrolase/transferase (1 copy) |

Table S5: Gaussian linear regression model results from testing which covariates significantly describe gut microbiome subtype groupings. P-values (alpha = 0.05) show an asterisk for significant and period for nearly significant values.

| **Variable** | **Estimate** | **Standard Error** | **P-value** |
| --- | --- | --- | --- |
| age | 4.78E-02 | 1.39E-02 | 0.0186 * |
| race: Asian | 3.73E+00 | 1.528 | 0.0587 . |
| race: MTOR | 4.375 | 1.599 | 0.041 * |
| race: White | 3.289 | 1.63 | 0.0996 . |
| weight (lb) | -1.47E-02 | 4.34E+01 | 0.0776 . |
| Alcohol (g) | -1.24E+02 | 4.34E+01 | 0.0353 * |
| Total Soluble Fiber (g) | -1.54E+03 | 6.53E+02 | 0.0647 . |
| Disaccharides (g) | 2.44E+02 | 6.88E+01 | 0.0164 * |
| Manganese (mg) | 8.03E+02 | 3.15E+02 | 0.0512 . |

Figure S1: Enterotypes describe gut microbiome subtypes to an extent in the present study. A stacked bar chart shows taxa indicative of enterotypes in green, orange, and purple (those in the grey other category being taxa not indicative of enterotypes). The y-axis shows taxonomic proportions in each microbiome subtype grouping, A, B, or C, in the dataset.

Figure S2: Differentially abundant genera between microbiome groups A and B identified by ANCOM. Group C was excluded due to low sample size. Bars show the relative abundance of the two genera detected as differentially abundant (*Bacteroides* and the Christensenellaceae R-7 group) under conservative ANCOM thresholds (detection cutoff 0.7 with significant effect size), colored by pre-treatment microbiome group. Relative abundances are shown for ease of visualization of the ANCOM-detected taxa. The test itself operates on log-ratio-transformed abundances.
